# The etiology of congenital diaphragmatic hernia: the retinoid hypothesis 20 years later

**DOI:** 10.1038/s41390-023-02905-7

**Published:** 2023-11-21

**Authors:** Juan F. Garcia Rivas, Robin D. Clugston

**Affiliations:** 1 Department of Physiology, University of Alberta, Edmonton, AB Canada; 2 Women and Children’s Health Research Institute, Edmonton, AB Canada

## Abstract

**Abstract:**

Congenital diaphragmatic hernia (CDH) is a severe birth defect and a major cause of neonatal respiratory distress. Impacting ~2–3 in 10,000 births, CDH is associated with a high mortality rate, and long-term morbidity in survivors. Despite the significant impact of CDH, its etiology remains incompletely understood. In 2003, Greer et al. proposed the Retinoid Hypothesis, stating that the underlying cause of abnormal diaphragm development in CDH was related to altered retinoid signaling. In this review, we provide a comprehensive update to the Retinoid Hypothesis, discussing work published in support of this hypothesis from the past 20 years. This includes reviewing teratogenic and genetic models of CDH, lessons from the human genetics of CDH and epidemiological studies, as well as current gaps in the literature and important areas for future research. The Retinoid Hypothesis is one of the leading hypotheses to explain the etiology of CDH, as we continue to better understand the role of retinoid signaling in diaphragm development, we hope that this information can be used to improve CDH outcomes.

**Impact:**

- This review provides a comprehensive update on the Retinoid Hypothesis, which links abnormal retinoic acid signaling to the etiology of congenital diaphragmatic hernia.
- The Retinoid Hypothesis was formulated in 2003. Twenty years later, we extensively review the literature in support of this hypothesis from both animal models and humans.

## Introduction

Congenital Diaphragmatic Hernia (CDH) is a life-threatening birth defect with high mortality that affects 2–3 in 10,000 newborns.^1–3^ CDH is characterized by incomplete formation of the diaphragm and associated lung hypoplasia, contributing to severe neonatal respiratory distress.^4,5^ In high-resource settings, at least 1 in 5 newborns with CDH will not survive the neonatal period (~20–30% mortality), with outcomes significantly worse in low-resource settings (>90% mortality).^6–8^ While rare, there is a considerable cost incurred when providing newborns with CDH intensive care, requiring significant healthcare resources.^3,9–11^ This burden on the healthcare system and patient families is compounded when considering that over half of surviving CDH patients have complex long-term morbidity.^12–15^ To fully grasp the clinical picture of CDH’s impact, the reader is referred to the recent review by Zani and colleagues.^5^

The etiology of CDH is complex and remains largely unknown.^5,16^ Different considerations include the clinical presentation and type of diaphragm defect. For example, CDH can present as an isolated defect (~50–60% of cases) or complex CDH, where additional abnormalities are present.^1,17,18^ Moreover, CDH can be thought of as an umbrella term incorporating different types of diaphragm defect.^19,20^ This includes the most commonly occurring Bochdalek CDH that is characterized by a hole in the posterolateral diaphragm (~90–95% of cases), anterior holes through the foramen of Morgagni (Morgagni hernias, ~3% of cases), diaphragm eventrations (~2–3% of cases), and central tendon defects (~1–2% of cases).^19,21,22^ As discussed below, it is important to consider that etiologic factors may vary between these different presentations of CDH. It is generally considered that the two main factors that contribute to CDH are genetic and/or environmental. Approximately 30–40% of CDH cases have an identifiable genetic cause.^3,23^ Of this number, chromosomal defects account for about ~10% of CDH cases, and de novo mutations account for 10–22% of cases.^24–26^ Environmental risk factors for CDH are varied, including associations with maternal age, alcohol use, and smoking.^3,16,27–30^ As the major focus of this review, one of the leading hypotheses to explain the etiology of CDH is the so-called Retinoid Hypothesis. This hypothesis was crystallized in the landmark paper published by Greer, Babiuk, and Thebaud in 2003, stating that “abnormalities linked with the retinoid signaling pathway early in gestation may contribute to the etiology of CDH”.^31^ The Retinoid Hypothesis is centered on the importance of the signaling molecule retinoic acid (RA) as a potent regulator of mammalian development.^32–34^ RA is an active metabolite of dietary vitamin A; the reader is directed to the following reviews that comprehensively describe its complex metabolism and signaling pathway.^35–37^

The 2003 formulation of the Retinoid Hypothesis was a milestone in CDH research and influenced subsequent work in the field.^31^ Here, we review discoveries that complement and strengthen the Retinoid Hypothesis, drawing upon work from both animal models and human CDH published in the last 20 years. Note, we have focused on RA signaling in abnormal diaphragm development; while there is an extensive literature linking RA, the lungs, and CDH, it is beyond the scope of this review.

## History of the Retinoid Hypothesis

The Retinoid Hypothesis was based on research dating back to the 1940s,^38^ drawing together several threads of evidence to justify the notion that altered retinoid signaling may cause CDH.^31^ The foundation for the Retinoid Hypothesis included evidence from animal models of CDH, as well as emerging clinical data. In brief, these studies included: (1) the observation that the offspring of vitamin A deficient rats had a high incidence of diaphragmatic defects, and when vitamin A was re-introduced into the diet, the rate of herniation decreased.^39–41^ (2) Compound Retinoic acid receptor (*Rar*) knock-out mice have a low incidence of CDH.^42,43^ (3) Nitrofen, a teratogen widely used to study CDH in rodents, inhibits the RA synthesizing enzyme RALDH2 (also called ALDH1A2) in vitro,^44^ and suppresses activation of a *Rar* reporter gene (*RARE-LacZ*) in vivo.^44,45^ (4) Nitrofen-induced CDH in rats can be reduced by the co-administration of large doses of vitamin A.^46^ And (5) in a small cohort of infants with CDH, circulating levels of retinol and its carrier protein in the blood (RBP) were decreased by ~50% in comparison with controls.^47^ The balance of this evidence led the authors to conclude that altered retinoid signaling may be a contributing factor in the etiology of CDH. As reviewed below, the formulation of the Retinoid Hypothesis had a significant impact on the field and its direction of research.

## New insights from animal models of CDH

Animal models of CDH are an important tool in improving our understanding of how CDH develops and its causes. Recent discoveries have helped to define the RA signaling pathway in the developing diaphragm, as well as provide important new insight into the role of vitamin A and its derivatives in the development of CDH gleaned from teratogenic and genetic models.

### Defining the retinoic acid signaling pathway in the developing diaphragm

**Fig. 1:**
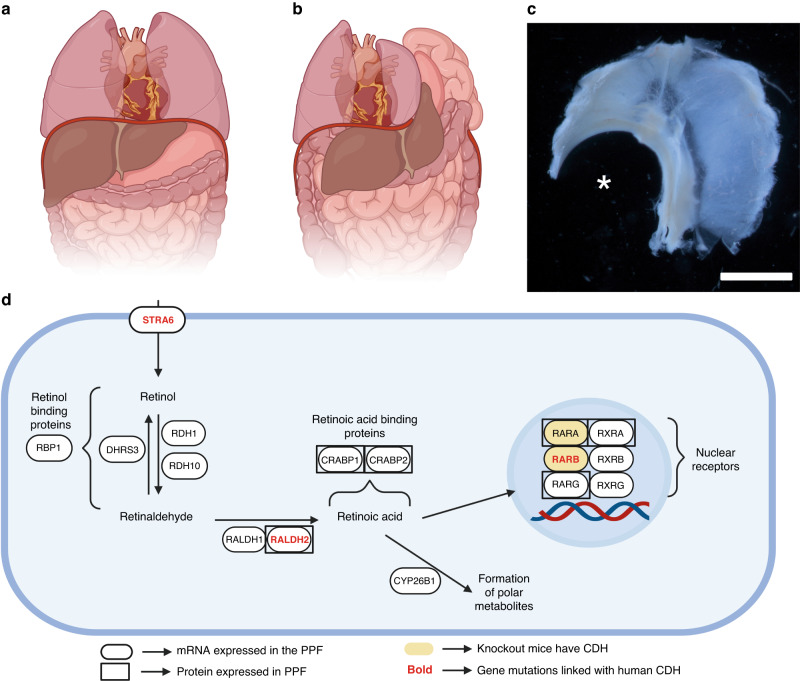
Congenital diaphragmatic hernia and retinoic acid signaling. The diaphragm forms an anatomical barrier between the abdominal and thoracic cavity (**a**). CDH is characterized by incomplete diaphragm development and herniation of abdominal organs into the thoracic cavity (**b**). Rodent models are widely used to models to study CDH. Here, a superior view of a whole diaphragm dissected from a teratogen-treated mouse fetus shows a large left-sided posterolateral (Bochdalek) diaphragm defect (asterisk; **c**). Retinoic acid signaling is thought to be essential for normal diaphragm development. A schematic representing our current knowledge regarding retinoic acid metabolism and signaling in the pleuroperitoneal fold (PPF), a key structure in the developing diaphragm is presented (**d**). This figure reflects known expression of specific genes and proteins in the PPF, as well as instances where mouse or human gene mutations have been associated with CDH. **c** scales bar = 500 µm. **a**, **b**, and **d** created with BioRender.com.

As the Retinoid Hypothesis has developed, it has become increasingly important to define the RA signaling pathway in the developing diaphragm, including identifying which specific components of the pathway are expressed in the developing diaphragm, and in which specific cells. Given our knowledge of CDH pathogenesis, this work has primarily focused on the pleuroperitoneal folds (PPFs); key structures in the developing diaphragm that are known to be disrupted in animal models of CDH.^48,49^ Immunohistochemical and transcriptomic studies have helped to define the RA signaling pathway in the PPF,^23,50^ with additional insight gained from other studies.^51^ Figure 1 describes our current understanding of the RA signaling pathway in the PPF, although we recognize that this pathway does not discriminate between different cellular subtypes within this structure, an important knowledge gap in the field given the suspected importance of specific cell types in the cellular origins of CDH, a topic beyond the scope of the current review.^49,52^

### Teratogenic models of CDH

Key studies in the original formulation of the Retinoid Hypothesis were based on data generated using the nitrofen model of CDH in rodents.^53–55^ While much of the early work used rats, mice are increasingly being used. The timing of teratogen administration is similar in both species (i.e., ~gestational day 8–10); however, for unknown reasons mice seem to be more resistant to nitrofen treatment, necessitating the use of teratogenic combinations to generate a high incidence of CDH.^50,56,57^ Recent work has strengthened the Retinoid Hypothesis by exploring how nitrofen produces CDH through the disruption of RA signaling, how exogenous retinoids can rescue teratogen-induced CDH, and how direct targeting of RAR signaling can causes CDH.

#### Mechanisms of teratogen-induced CDH

The Retinoid Hypothesis was founded on reports that nitrofen can inhibit RALDH2 and suppress activation of RAR signaling.^44,45^ Noble and colleagues conducted a series of mechanistic studies to better understand how nitrofen disrupted retinoid signaling to induce CDH.^58^ This work provided further evidence that nitrofen inhibits RALDH2 using a *RARE-LacZ* reporter system in P19 cells, which could be overcome by adding excess enzyme substrate in the form of retinal.^58^ It was subsequently shown in rats that nitrofen treatment on gestational day 9 significantly decreased fetal RA levels between gestational day 11 and 13, which coincides with a critical time in diaphragm development and provided unequivocal evidence that nitrofen treatment could inhibit RA synthesis in vivo.^58^ This decrease in RA synthesis complemented the previously observed decrease in activation of the *RARE-LacZ* reported gene in nitrofen-treated mice.^45^ Indeed, nitrofen and three other CDH-inducing teratogens that inhibit RALDH2 all decrease embryonic retinoid acid signaling throughout the critical period of diaphragm development, as subsequently shown in *RARE-LacZ* mice.^50^ While alternative mechanisms have been suggested,^59,60^ the evidence suggests that nitrofen primarily induces CDH by inhibiting RALDH2, thereby decreasing RA synthesis and inhibiting downstream signaling through the RARs.

#### Exogenous retinoids can rescue teratogen-induced CDH

Powerful evidence to support nitrofen’s mechanism of action and the importance of retinoid signaling in diaphragm development came from the observation that administration of vitamin A could lower the incidence of nitrofen-induced CDH in rats.^46^ Using a dose of 15,000 IU of vitamin A, Thebaud et al. decreased the incidence of nitrofen-induced CDH by over half.^46^ Using the same dose of vitamin A, other groups confirmed that co-administration of vitamin A could lower the incidence of nitrofen induced CDH in rats, while also showing beneficial effects of exogenous vitamin A on the developing lung.^61,62^ Based on the model that nitrofen inhibits RA synthesis, subsequent studies tested the hypothesis that administration of RA would be more efficacious than vitamin A (retinol) administration in preventing teratogen-induced CDH.^63^ These studies showed that while vitamin A administration (25,000 IU vitamin A) reduced the incidence of CDH by ~40%, RA had a more potent rescue effect, lowering the incidence by ~80%.^63^ The potent effect of direct supplementation with RA to prevent CDH was further emphasized in teratogen-treated mice.^50^ Here, a high incidence of CDH (~60% CDH) was almost completely abrogated by co-administration with RA (~1% CDH), with the authors further showing that while teratogen treatment decreased embryonic *RARE-lacZ* expression, RAR signaling was restored by RA treatment in accord with the decrease in CDH incidence.^50^

A limitation of the rescue studies discussed above was the high dose of vitamin A (e.g., 15,000–25,000 IU vitamin A) required to reverse the effects of nitrofen. Interestingly, based on the observation that low maternal dietary vitamin A intake is a risk factor for developing CDH in humans,^64,65^ it has been shown that altered dietary vitamin A intake can modulate the incidence of teratogen-induced CDH, such that mice consuming a vitamin A deficient diet have a higher incidence of CDH.^57^ This study is important because it shows that high, pharmacological doses of vitamin A are not required to prevent teratogen-induced CDH, rather, it can be achieved through increased dietary vitamin A intake. As discussed below, this is important in the context of low maternal dietary vitamin A intake in humans as a potential risk factor for CDH.

Taken together, several studies have shown the efficacy of high doses of vitamin A to prevent teratogen-induced CDH, with more recent work suggesting that adequate dietary vitamin A intake can achieve a similar result. These studies highlight the importance of RA signaling in the development of teratogen-induced CDH and the feasibility of preventing CDH, at least in animal models.

#### Directly targeting retinoic acid receptor signaling induces CDH

As discussed above, there is strong evidence to suggest that nitrofen induces CDH by inhibiting RA synthesis via RALDH2. Other potential mechanisms of nitrofen’s action have been suggested,^58–60^ representing a potential limitation in the interpretation of these studies. On the other hand, studies using the pan-RAR antagonist BMS493 provide direct evidence in support of the Retinoid Hypothesis, showing that specific inhibition of RA signaling is a potent inducer of Bochdalek CDH in rats.^50^ Indeed, a 5 mg/kg dose of BMS493 induced almost 100% herniation when given to pregnant dams on gestational day 11. BMS493 administration was further used to define the critical period of diaphragm development in rats, which spanned gestational days 8 to 13. Remarkably, separate critical periods were identified for the left and right hemi-diaphragms, with the left side of the diaphragm being preferentially affected with BMS493 administration between gestational days 8 and 9, the right side preferentially affected between gestational days 11 and 13, and bilateral hernias produced with administration on gestational day 10. Thus, direct targeting of RAR signaling on different gestational days induced CDH in a temporally and spatially restricted manner.^50^ While BMS493 was first used to induce CDH in rats, subsequent research has shown that this compound can also induce CDH in mice, albeit at a lower incidence (~6%).^66^ Similarly, <1% of mice treated with the pan-RAR antagonist BMS-189453 also developed CDH.^67^

### Genetic models of CDH

The observation that *Rar* knock-out mice developed CDH was integral to the original formulation of the Retinoid Hypothesis.^31^ Surprisingly, there has been a lack of studies genetically dissecting the RA signaling pathway in the developing diaphragm; however, several other mouse models with an indirect relationship to RA signaling have been described that are supportive of the Retinoid Hypothesis.

#### Genetic models directly related to retinoic acid signaling

The only direct genetic evidence supporting the Retinoid Hypothesis obtained from mice with targeted mutations in RA signaling come from studies of *Rar* compound mutant mice (Fig. 1). There are three *Rars* encoded in the mammalian genome, *Rara*, *Rarb*, and *Rarg.*^32,68^ Single knock outs for each *Rar* do not produce diaphragm defects; however, a low incidence of CDH has been reported in *Rara*^*−/−*^*:Rarb*^*−/−*^ compound mutant mice, which was not observed in *Rara*^*−/−*^*:Rarg*^*−/−*^ or *Rarb*^*−/−*^*:Rarg*^*−/−*^compound mutants.^42,43^ Interestingly, diaphragm defects were also reported in *Rxra* transgenic mice that lack their activation function domain (AF-2), highlighting the importance of this heterodimeric partner of the RARs.^69^ To our knowledge, there are no other published studies describing diaphragm defects in mice carrying mutations in genes directly associated with RA signaling. Indeed, many knock-out mice in this pathway are grossly normal and viable (e.g., *Lrat, Crbp1*, and *Rbp4*).^70–72^ The lack of a diaphragm phenotype in these mice may reflect robustness/compensation in the RA signaling pathway, or evidence that they are not essential in diaphragm development. Conversely, some knock-out mice in this pathway are embryonic lethal (e.g., *Raldh2, Cyp26a1*, and *Dhrs3*)^73–75^ precluding an assessment of diaphragm formation. In this latter case, experimental approaches using conditional deletion of specific genes could allow testing of their hypothetical importance in the developing diaphragm.

#### Genetic models indirectly related to retinoic acid signaling

In addition to *Rar* compound mutant mice, diaphragm defects have been described in other genetic models, including several genes indirectly linked to RA signaling.^76^ This link is most clear with *Wt1*, which encodes a transcription factor with a broad expression pattern, including the PPF.^77–79^ The original description of *Wt1*^−/−^ mice emphasized its role in urogenital development but also mentioned diaphragm defects that were later found to be like Bochdalek CDH.^80,81^ Because of its broad importance in embryonic development,^82^ *Wt1*^*−/−*^ mice typically do not survive into late gestation, limiting the ability to study diaphragm development.^81^ This limitation was overcome by conditional deletion of *Wt1*. Carmona et al. used an enhancer of *Gata4* to conditionally delete *Wt1* in the lateral plate mesoderm, leading to a ~80% incidence of Bochdalek CDH.^51^ Similarly, Cleal et al. conditionally deleted *Wt1* in the PPF to generate offspring with an 80–90% penetrance of Bochdalek CDH.^77^ Regarding RA signaling, WT1 regulates the expression of the RA synthesizing enzymes RALDH2,^83^ and *Wt1* itself is thought to be a RA target gene.^84^ Interestingly, dietary RA supplementation has been shown to relieve the incidence of CDH in mice with conditional Wt1 deletion and decrease the size of diaphragmatic defects in embryos that still displayed CDH.^51^

Like WT1, COUP transcription factor 2 (COUP-TF2, encoded by *Nr2f2*) is a developmentally important transcription factor with links to RA signaling.^85,86^ It has been shown that COUP-TF2 is upregulated by RA, and it can regulate gene expression by modulating the dimerization of RAR/RXRs.^84,87–90^ Global *Coup-tf2*^*−/−*^ mice are embryonically lethal; however, conditional deletion of *Coup-tf2* in the PPF induces Bochdalek CDH in ~50% of offspring.^91^ Two other genes that encode transcription factors that functionally interact with each other and have been linked to RA signaling and CDH are *Gata4* and *Fog2* (also known as *Zfpm2*). GATA4 is involved in many different developmental processes, the most important one being proper cardiac development.^92–94^ *Gata4*^*−/−*^ mice are embryonic lethal, but mice heterozygous for a Gata4 mutation (exon 2 deletion) develop midline diaphragm defects, impacting the central tendon in ~30% of offspring and causing overt herniation in ~15% of offspring.^95^ Furthermore, conditional deletion of *Gata4* in the PPF caused diaphragm defects in 100% of mutant mice, 2/3 of which were Bochdalek hernias, and the remaining 1/3 Morgagni hernias.^96^ *FOG2* is thought of as a co-factor of the GATA transcription factors and has been shown to interact with GATA4 in heart development.^97^ Mice with a mutation in *Fog2* present diaphragm abnormalities characterized by incomplete muscularization of the posterolateral diaphragm in 100% of mutant mice.^98^ Regarding the Retinoid Hypothesis, both *Gata4* and *Fog2* are thought to be RA target genes,^84,87,99,100^ and it has been shown that retinoids can regulate downstream gene expression by interacting with GATA4 and FOG2.^101^ Interestingly, FOG2 has also been shown to interact with COUP-TF2, suggesting complex interactions with multiple transcription factors that are important in diaphragm development.^102^

The presence of diaphragm defects in *Wt1*, *Coup-tf2*, *Gata4*, and *Fog2* mutant mice indicates their individual importance in normal diaphragm development. Links between these genes and RA signaling support the Retinoid Hypothesis, as recently discussed by others.^103^ Future studies integrating our understanding of how these genes interact should provide insight into the cellular pathogenesis and etiology of CDH.

### New Insights from human cases of CDH

When Greer and colleagues formulated the Retinoid Hypothesis, there was sparse evidence linking it with CDH in infants.^31^ This represented a limitation of the hypothesis; however, in the intervening 20 years new insights driven by advances in clinical genetics, as well as epidemiological studies have strengthened the relevance of the hypothesis to CDH in humans.

#### Retinoid status of infants with CDH

The only clinical data in support of the Retinoid Hypothesis when it was formulated came from a small study of vitamin A status in 11 cases of CDH.^31,47^ This study found that newborns with CDH had a ~50% decrease in circulating retinol and retinol binding protein (RBP) concentrations compared to healthy controls.^22^ A follow-up study including 22 newborns with CDH confirmed this observation, reporting significantly lower retinol and RBP concentrations in newborns with CDH compared to matched controls; however, the magnitude of this decrease was relatively smaller (~20–25% decrease).^104^ Neither study found a decrease in maternal markers of vitamin A status, suggesting that maternal vitamin A deficiency was not a contributing factor to CDH in these patient populations. Thus, these two studies suggest that the vitamin A status of newborns with CDH is somehow impaired, although the reason for this remains elusive and requires further study. To our knowledge, there has been no other comprehensive examination of vitamin A status in CDH. One autopsy study including nine cases of CDH attempted to indirectly measure retinoid stores in the liver and lung via CRBP1 immunoreactivity.^105^ These results were interpreted in the context of “retinoic acid status”, which we have questioned as incorrect,^106^ nevertheless, this study hints at the possibility of impaired vitamin A status of fetuses with CDH. A physiological decline in circulating maternal retinol levels has been reported in human pregnancy, with a rebound to non-pregnant levels after birth.^107–109^ A similar pattern is seen in pregnant mice and rats.^110,111^ This has led to the suggestion that these declines correspond with a critical period in diaphragm development and may render this structure more susceptible to aberrant RA signaling.^31,103^ Variations in circulating maternal retinol levels, fetal retinoid status, and their importance in CDH require further study.

#### The genetics of CDH and the Retinoid Hypothesis

The genetic etiology of CDH is complex and continues to be an active area of research and discovery.^16,76,112^ When the Retinoid Hypothesis was first proposed, our understanding of the genetics of CDH was limited; however, since then, there has been progress in understanding the genetic etiology of CDH, as well as how this new knowledge relates to the Retinoid Hypothesis.^103,113^ Like the mouse genetics, two themes emerge from the human genetics of CDH in relation to the Retinoid Hypothesis: a subset of genes associated with CDH are directly involved in RA signaling, while others can be indirectly linked to RA signaling either as target genes or factors that potentially interact with RAR signaling.

#### Human genetic disorders directly linking retinoid acid signaling and CDH

Goumy et al. systematically discussed the RA signaling pathway and drew links of varying strength with several CDH-associated genes.^113^ The strongest evidence linking RA signaling and CDH in humans exists for *STRA6*, *RALDH2*, and *RARB* (Fig. 1). *STRA6* (OMIM: 610745) encodes the membrane receptor for RBP, which acts as a transporter for the cellular uptake of retinol.^114^ *STRA6* mutations cause Matthew Wood syndrome, which includes microphthalmia, pulmonary hypoplasia, cardiac malformations, as well as diaphragm defects variously reported as CDH/diaphragm eventration in ~25% of cases.^115–117^ Diaphragm defects are frequently associated with *STRA6* mutations, and this gene is known to be expressed in the developing diaphragm, strongly linking *STRA6* with CDH.^23,118^ *ALDH1A2 (RALDH2*; OMIM: 603687) encodes a protein which synthesizes RA.^119^ Mutations in this gene cause a variety of different phenotypes, including tetralogy of Fallot, absent thymus, kidney defects, as well as diaphragm eventration/Bochdalek CDH.^120,121^ Four known patients with mutations in this gene have diaphragm defects, it is expressed in the developing diaphragm, and its inhibition causes CDH in rodents, strongly linking *RALDH2* with CDH.^23,44,58,120–122^ *RARB* (OMIM: 180220) encodes for the β isoform of the RAR, a nuclear receptor activated by RA that regulates gene expression.^123^ Mutations in this gene are associated with diaphragmatic hernias as well as eventrations.^124–126^ *RARB* mutations have been reported in 9 patients with CDH, its mutation is linked with CDH in mice, and it is expressed in the developing diaphragm, strongly linking it with CDH.^124,125^ As discussed by Goumy et al. and others, *RBP1*, *RBP2*, *RBP5*, and *LRAT* have all been linked with CDH, although the evidence is relatively weak.^113,127^ These links are primarily driven by the proximity of these genes to chromosomal regions recurrently deleted in individuals with CDH, but to our knowledge no specific gene mutations have been described in them directly linking them to CDH.

#### Human genetic disorders indirectly linking retinoid acid signaling and CDH

Genes that have been linked with CDH in humans that are indirectly involved in RA signaling are numerous and can be broadly categorized as genes whose protein products interact with RAR function or they are RA target genes.

As discussed above, there is significant evidence linking genes encoding proteins that interact with RAR function to CDH in animal models, including *Wt1*, *Coup-tf2*, *Gata4*, and *Fog2*. Interestingly, all these genes are also associated with CDH in humans. Mutations in *WT1* (OMIM: 607102) are associated with several syndromes that include diaphragm defects within their phenotypic spectrum, including Denys-Drash syndrome, Frasier syndrome, and Meacham syndrome.^128^ Amongst several other genes, *COUP-TF2* (OMIM: 107773) is located within the 15q26.1–26.2 cytogenetic hotspot for syndromic CDH.^129^ The occurrence of CDH in mice with a conditional deletion of *Coup-tf2* and the later discovery of specific mutations in this gene in individuals with CDH support its importance in diaphragm development.^91,130^ *GATA4* (OMIM: 600576) is found within the CDH critical region 8p23.1,^127^ with subsequent reports of *GATA4* variants in humans causing CDH.^131^ Similarly, *FOG2* (OMIM: 603693) was originally linked to CDH as it is in the critical region 8q22-8q23,^127^ with later studies showing specific mutations/deletions of this gene were recurrently associated with CDH.^98,132^ *WT1*, *COUP-TF2*, *GATA4,* and *FOG2* are all expressed in the developing diaphragm, and all are strongly linked to CDH via both mouse and human mutations. All four genes have also been linked to RA signaling, providing supporting evidence for the importance of this pathway in normal diaphragm development.

Interestingly, *COUP-TF2*, *GATA4,* and *FOG2* are all linked to RA signaling via interactions with the RARs. While the evidence is less strong, there are other genes that can be linked to RA signaling in this way. Predicted pathogenic variants in *NSD1* have been identified in a cohort of fetuses with CDH.^133^ This gene is also associated with Beckwith-Wiedemann syndrome that infrequently includes diaphragm defects.^133–135^ Moreover, *NSD1* is another protein that can interact with nuclear receptors, including the RARs.^136^ Similarly, while there has only been one known report linking it with CDH, *SIN3A* encodes a transcriptional corepressor that interacts with RARs.^26,137^ Last, while its exact interaction with RAR signaling remains unclear, *KIF7* and RA signaling have been linked to the development of diaphragm defects in mice and three cases of CDH.^138–140^

In addition to genes that may interact with RA signaling at the level of the receptor, diaphragm defects have been described in association with mutations in numerous RA target genes. In our past analysis of CDH-associated genes, we identified an inclusive list of 218 genes linked with CDH, 44 of which were RA target genes.^21^ This category includes genes regulated by RA that have direct roles in RA signaling (e.g., *STRA6*, *RALDH2*, and *RARB*), and indirect interactions with RA signaling (e.g., *WT1*, *COUP-TF2*, *GATA4*, and *FOG2*). Thus, it is possible to envisage how aberrant RA signaling in the developing diaphragm could impact the expression of multiple genes linked with diaphragm development and precipitate CDH.

As we learn increasingly more about the genetics of CDH, a common pathway that has emerged is RA signaling, providing support for the Retinoid Hypothesis in human CDH.^21^ As discussed above, many CDH-associated genes can be linked with RA signaling, the challenge for the future will be to determine how these genes contribute to diaphragm development and whether it is possible to construct a gene regulatory network to show the relationship between these genes and how aberrant RA signaling can lead to abnormal diaphragm development.

#### Epidemiological studies and the Retinoid Hypothesis

Population-based studies have provided insight into the link between CDH and the Retinoid Hypothesis, primarily in the context of adequate maternal dietary vitamin A intake. One of the first epidemiological studies to link vitamin A intake and CDH leveraged maternal nutrient intake data between 1997 and 2003 from the US National Birth Defects Prevention Study.^141^ Vitamin A intake below the 10th percentile was associated with isolated CDH in women who did not use periconceptional vitamin supplements (retinol intake, odds ratio [95% CI] = 2.1 [1.1, 3.9]), and in women who did use periconceptional vitamin supplements (total vitamin A intake, odds ratio [95% CI] = 1.7 [1.2, 2.6]). The authors concluded that this data supported the Retinoid Hypothesis, but it is important to note that an expanded analysis of the US National Birth Defects Prevention Study including maternal nutrient intake data from 1997 to 2011 did not find a significant association between vitamin A intake and CDH.^142^ Despite this lack of agreement, two other studies from The Netherlands and Japan have shown that low maternal vitamin A intake confers an increased risk of CDH.^64,65^ In a Dutch population, Beurskens et al. showed that maternal dietary vitamin A intake below the recommended daily intake (<800 µg vitamin A per day) in normal weight mothers was significantly associated with an increased risk of CDH (odds ratio [95% CI] = 7.2 [1.5, 34.4]). In a Japanese population, Michikawa et al., showed that in a similar group of normal weight mothers, high total maternal dietary vitamin A intake was associated with a reduced risk of CDH (odds ratio [95% CI] = 0.6 [0.3, 1.2]). Taken together, there is collective epidemiological evidence that low maternal dietary vitamin A intake is a risk factor for CDH, although this concept requires further exploration.

While there is evidence to suggest that maternal dietary vitamin A intake is an important factor in the etiology of CDH, it has been observed that there is no evidence to suggest an increased incidence of CDH in countries with high rates of vitamin A deficiency.^104^ A counterpoint to this argument is that birth defect registries in these countries are inadequate, and data regarding the global incidence of CDH is incomplete.^3^ While considering maternal dietary vitamin A intake as a risk factor for CDH it is important to highlight that even in developed countries inadequate dietary intake can be prevalent in the general population and has been reported in 15.5% of pregnancies in the USA (*n* = 1003), and 10% in Poland (*n* = 1764).^143,144^ In a separate study, ~7% of women of childbearing age in the USA had low serum retinol concentrations, a factor that was linked to lower socioeconomic status.^145^ While overt vitamin A deficiency might not be a major contributor to the occurrence of CDH, we believe inadequate dietary vitamin A intake is a risk factor that may combine with other factors (genetic or environmental) to cause CDH.^57^ Indeed, we echo the recent remarks made by Gilbert and Gleghorn,^103^ supporting adequate maternal vitamin A intake during pregnancy at the population level to help prevent CDH, and possibly other birth defects.

Regarding other environmental risk factors that may intersect with the Retinoid Hypothesis, it is interesting to note that others have drawn links between maternal alcohol use and cigarette smoking and altered retinoid signaling.^146^ While these links are speculative and require further study, there is experimental evidence that links both alcohol exposure and cigarette smoke exposure to alterations in vitamin A metabolism.^146–148^

### Future perspectives

Looking forward, it is important to consider the limitations of the Retinoid Hypothesis. There are many gaps in our knowledge regarding the hypothesis and here we pose several questions aimed at guiding future studies in the field.

#### How does the Retinoid Hypothesis explain abnormal diaphragm development at a cellular level?

There is an incomplete understanding of the cellular pathogenesis of CDH. It is thought that the non-muscular mesenchymal cells of the PPF are important in the development of CDH,^56,122,149^ but how RA signaling works in these cells, and the fate of these cells when RA signaling is disrupted, remains unexplored. Future studies using animal models of CDH can address these gaps, supplemented by studies in PPF-derived cell cultures, and patient-derived fibroblasts.^150,151^

#### Does the Retinoid Hypothesis explain all types of diaphragm defects?

The evidence supporting the Retinoid Hypothesis primarily comes from models recapitulating Bochdalek CDH. Moreover, past analysis of CDH-associated genes identified a link between genes involved in retinoid signaling and Bochdalek CDH, but not other types of diaphragm defects.^21^ Thus, while the Retinoid Hypothesis helps explain the etiology of Bochdalek CDH, there is less evidence linking it to diaphragm eventration, central tendon defects, and Morgagni hernias. This gap in understanding the etiology of rare subtypes of CDH should be the focus of future research.

#### Does the Retinoid Hypothesis explain all cases of CDH?

The percentage of CDH cases caused by abnormal retinoid signaling is unclear and difficult to estimate. In terms of CDH genetics, it is clear from relatively large cohort studies that genetic mutations directly linked to RA signaling are uncommon.^152,153^ In our previous analysis of 218 CDH-associated genes, only 11 were directly related to RA signaling.^21^ Interestingly, 52 of these genes could be indirectly linked as modulators of RA signaling or as RA target genes. If we consider population-based studies associating low maternal dietary vitamin A intake and CDH,^64,65^ then gene-nutrient interactions impacting RA signaling may emerge as a major contributor to the etiology of CDH. Nevertheless, as discussed elsewhere, alternate explanations for the etiology of CDH exist.

#### What are the alternative hypotheses to explain the etiology of CDH?

Previous links between CDH and altered thyroid hormone signaling and one-carbon metabolism, have been suggested but not supported by further analyses.^58,141,142,154^ Other alternative hypotheses that remain to be fully explored include a possible role for maternal dietary vitamin D intake,^155,156^ as well as an emerging interest in extracellular vesicles and micro RNAs.^5,157^

#### Does the Retinoid Hypothesis link abnormal diaphragm and lung development?

While beyond the scope of the current review, it is important to acknowledge that RA signaling is important in lung organogenesis.^136–138^ In 2000, Keijzer et al. proposed the Dual-hit Hypothesis to explain pulmonary hypoplasia in CDH, which included a direct effect on the lung (first hit) and indirect effects via physical compression secondary to herniation (second hit).^139^ Given RA’s role in lung development, and its emerging role in the diaphragm, the Retinoid Hypothesis is consistent with a dual-hit model of lung damage in CDH. Here, perturbed RA signaling could contribute to abnormal lung development (first hit), which would be compounded by the effects of perturbed RA signaling on diaphragm formation leading to abdominal organ herniation and further damage to the lungs (second hit), as explored by others.^140–142^ Whether altered RA signaling contributes to both abnormal lung and diaphragm development in CDH requires further study.

#### How does the Retinoid Hypothesis relate to CDH in humans?

The strongest evidence for the Retinoid Hypothesis comes from animal models. To strengthen the link with human CDH there should be a continued emphasis on studying the importance of maternal dietary vitamin A intake as a risk factor for CDH, more attention paid to markers of maternal and fetal vitamin A status in CDH, and a continued exploration of interactions between RA signaling and CDH-associated genes.

#### How do we leverage our understanding of the Retinoid Hypothesis to lessen the impact of CDH?

Zani et al. recently emphasized that by improving our understanding of CDH’s etiology we may improve its diagnosis and allow earlier interventions to improve CDH outcomes.^5^ Retinoid administration is already being studied as a tool to improve lung development in preclinical models of CDH,^158–160^ which may be optimized by improving our understanding of underlying defects in RA signaling. Furthermore, if early genetic testing revealed mutations in genes linked with RA signaling, it might be possible to intervene with retinoids to help ameliorate damage to the developing diaphragm/lungs, lessening the impact of CDH. At the population level, we and others believe that attaining optimal maternal dietary vitamin A intake may help lessen the impact of CDH.^57,103^ This would require efforts highlighting the importance of adequate periconceptional vitamin A intake in women, and their potential benefits with respect to CDH.

### Concluding remarks

In the 20 years since the Retinoid Hypothesis was first proposed many studies across multiple disciplines have further supported the relationship between RA signaling and the formation of CDH.^31^ While the Retinoid Hypothesis has a strong evidence base derived from animal models and growing support from clinical studies, it does have limitations and gaps in our knowledge remain that should guide future research in the field, hopefully translating to improved outcomes for newborns with CDH.
